# Deletion of ErbB4 Disrupts Synaptic Transmission and Long-Term Potentiation of Thalamic Input to Amygdalar Medial Paracapsular Intercalated Cells

**DOI:** 10.3389/fnsyn.2021.697110

**Published:** 2021-07-28

**Authors:** Douglas Asede, James Okoh, Sabah Ali, Divyesh Doddapaneni, M. McLean Bolton

**Affiliations:** Disorders of Neural Circuit Function, Max Planck Florida Institute for Neuroscience, Jupiter, FL, United States

**Keywords:** ErbB4, inhibition, excitation, amygdala, intercalated amygdalar cells, fear circuit, LTP

## Abstract

Identification of candidate risk genes and alteration in the expression of proteins involved in regulating inhibitory neuron function in various psychiatric disorders, support the notion that GABAergic neuron dysfunction plays an important role in disease etiology. Genetic variations in neuregulin and its receptor kinase ErbB4, expressed exclusively by GABAergic neurons in the CNS, have been linked with schizophrenia. In the amygdala, ErbB4 is highly expressed in GABAergic intercalated cell clusters (ITCs), which play a critical role in amygdala-dependent behaviors. It is however unknown whether ErbB4 deletion from ITCs affects their synaptic properties and function in amygdala circuitry. Here, we examined the impact of ErbB4 deletion on inhibitory and excitatory circuits recruiting medial paracapsular ITCs (mpITCs) using electrophysiological techniques. Ablation of ErbB4 in mpITCs suppressed NMDA receptor-mediated synaptic transmission at thalamo-mpITC synapses and enhanced thalamic driven GABAergic transmission onto mpITCs. Furthermore, long-term potentiation (LTP) at thalamo-mpITC synapses was compromised in ErbB4 mutant mice, indicating that ErbB4 activity is critical for LTP at these synapses. Together, our findings suggest that ErbB4 deletion from mpITCs disrupts excitation-inhibition balance and learning mechanisms in amygdala circuits.

## Introduction

The amygdala plays a critical role in acquisition and storage of fear memory and other social-emotional-related behaviors (LeDoux, 2000, 2007; Felix-Ortiz and Tye, 2014; Felix-Ortiz et al., 2016). The interactions between excitatory and inhibitory neurons play a critical role in coordinating network activity during different behavioral states (Li et al., 2013; Penzo et al., 2014; Wolff et al., 2014). The neuronal composition of the basolateral structures of the amygdala (BLA) is similar to that of the cortex; consisting of a majority of glutamatergic projection neurons and a minority of local GABAergic interneurons (Ehrlich et al., 2009). The central nucleus of the amygdala (CeA) is striatum-like, with majority of GABAergic neurons. In addition to these nuclei is a network of interconnected GABAergic neurons organized in clusters surrounding the BLA known as the intercalated cell clusters (ITCs). These neurons express the dopamine D1 receptor (Drd1) (Fuxe et al., 2003; Palomares-Castillo et al., 2012) and are required for fear expression and extinction (Likhtik et al., 2008; Busti et al., 2011). In particular, neurons in the medially located cluster (mpITC) receive fear-related sensory thalamic drive and exert strong inhibitory control on BLA and CeA output thereby regulating amygdala-dependent behaviors (Royer and Pare, 2002; Manko et al., 2011; Huang et al., 2014; Asede et al., 2015). This inhibitory influence is thought to be gated by the action of signaling peptides and neuromodulators; thus, internal state is relayed to the amygdala by the ITC network (Jungling et al., 2008; Blaesse et al., 2015; Kwon et al., 2015).

The development and function of synaptic connections is regulated by a myriad of molecular signaling cascades that alter the expression of synaptic proteins. Among these is the transmembrane receptor kinase for neuregulin (NRG) – ErbB4, which is exclusively expressed by GABAergic neurons in the CNS (Bean et al., 2014). Mutations in the ErbB4 gene have been linked with schizophrenia (Hahn et al., 2006; Walsh et al., 2008; Vullhorst et al., 2009; Bean et al., 2014; Mei and Nave, 2014). While several genetic studies have suggested a link between the NRG-ErbB4 signaling network and brain disorders such as schizophrenia, the pathophysiological mechanisms underlying this disease susceptibility are only starting to be explored in animal models (Mei and Nave, 2014). ErbB4 null mice and those with conditional deletion of ErbB4 from hippocampal fast-spiking PV interneurons exhibited a schizophrenia-like phenotype and had several synaptic deficits (Shamir et al., 2012; Del Pino et al., 2013). ErbB4 deletion from PV interneurons in the BLA resulted in impaired tone-cued and contextual fear conditioning, suggesting a role for ErbB4 in fear learning (Shamir et al., 2012; Lu et al., 2014; Chen Y. H. et al., 2017). During fear learning, BLA interneurons and ITCs exert spatiotemporal control over the activity of BLA principal neurons by dynamically regulating neuronal excitation in a cell-type and subcellular compartment-specific manner (Wolff et al., 2014; Asede et al., 2015; Letzkus et al., 2015; Krabbe et al., 2018). This raises the question of how ErbB4 expression affects the activity of GABAergic neurons in the amygdala. In the BLA, application of AG1478, an inhibitor of ErbB4 kinases reduced the amplitude of evoked inhibitory postsynaptic currents (IPSCs) recorded in BLA principal neurons, suggesting a role of ErbB4 kinases in maintaining inhibition in BLA (Lu et al., 2014). Although ErbB4 is highly expressed in the ITCs, its effect on synaptic function is unknown. In this study, we analyzed inhibitory and excitatory inputs that recruit mpITCs and provide novel evidence for the role of ErbB4 in maintaining mpITC function in amygdala fear circuits. We found that deletion of ErbB4 in mpITCs disrupts inhibitory transmission, NMDA receptor–mediated synaptic transmission, and long-term potentiation (LTP) of sensory inputs onto mpITCs.

## Materials and Methods

### Animals

To examine the effects of ErbB4 deletion on ITC function, we generated an ITC targeted ErbB4 knockout (ErbB4^F/F^:Drd1a^Cre(EY266)^; referred to as ErbB4 cKO) and controls (ErbB4^WT/WT^:Drd1a^Cre(EY266)^; referred to as WT) by crossing ErbB4 floxed mice (010439-UCD) with a Drd1a-CRE transgenic (EY266) (MMRRC 30779). In this line, CRE recombinase is expressed in a subset of D1R expressing neurons, which is mostly restricted to ITCs in the amygdala. Although this particular D1R-Cre line expresses Cre in a sparse, scattered subset of excitatory neurons and in some D1 medium spiny neurons (MSNs) in nucleus accumbens shell and amygdala-striatum transition zone, ErbB4 is neither expressed in excitatory neurons nor in most GABAergic neurons in the basal ganglia (Bean et al., 2014). In all electrophysiological recordings, to label and verify the identity of patch-clamped mpITC neurons in Drd1a^Cre(EY266)^ line, we crossed the mice with Ai9 mice (JAX, line 007902) that express tdTomato in the presence of Cre recombinase. Similarly, for identification in conditional knockout (cKO) mice, we crossed ErbB4^F/F^:Drd1a^Cre(EY266)^ mice with Ai9 mice.

### Viruses and Stereotactic Injections

For optogenetic stimulation of thalamic afferents in the amygdala, 5–7-week-old mice maintained under isoflurane anesthesia were stereotaxically injected with AAV1-hSyn-hChR2(H134R)-eYFP, titer 1.73E (Manko et al., 2011) (AV1-26973, PennVector Core, Philadelphia, PA, United States) into the posterior intralaminar nucleus/medial part of the medial geniculate nucleus (PIN/MGm) using the following coordinates from bregma (in mm): posterior −3.0, lateral ±1.8, and ventral −3.8. All virus injections were performed using Neurostar Robot stereotaxic systems with integrated brain atlas (Neurostar, Sindelfingen, Germany).

### Slice Preparation and Patch Clamp Recordings

Eight to twelve week-old mice were deeply anesthetized with isoflurane and then decapitated. The brain was rapidly removed and acute coronal brain slices (300 μm) were prepared in ice-cold cutting solution containing (in mM) (Asede et al., 2020): 124 choline chloride, 26 NaHCO_3_, 2.5 KCl, 3.3 MgCl_2_, 1.2 NaH_2_PO4, 10 glucose, and 0.5 CaCl_2_. After cutting, slices were allowed to recover for 30 min at 32°C and stored at room temperature in artificial cerebrospinal fluid (ACSF) containing (in mM): 124 NaCl, 26 NaHCO_3_, 3 KCl, 1.25 NaH_2_PO_4_, 20 glucose, 1 MgCl_2_, 2 CaCl_2_, 5 sodium ascorbate, 3 sodium pyruvate, and 2 thiourea. All solutions were continuously bubbled with 95% O_2_/5% CO_2_. Slices containing the amygdala were transferred to a submersion recording chamber, superfused with oxygenated ACSF at a speed of 1–2 ml/min. Whole-cell patch-clamp recordings were performed using pipettes pulled from borosilicate glass capillaries (BF150-110-10, Sutter Instrument, United States) with resistances of 6–8 MΩ. Postsynaptic currents were recorded in voltage clamp mode using Cs-methanesulfonate based internal solution containing (in mM): 135 Cs-methanesulfonate, 6 NaCl, 10 HEPES, 0.6 EGTA, 4 MgATP, and 0.3 Na_2_GTP. For current clamp experiments, K-gluconate based intracellular solution containing (in mM): 145 K-gluconate, 6 NaCl, 10 HEPES, 0.6 EGTA, 4 MgATP, and 0.3 Na_2_GTP was used. To study basal synaptic transmission from the thalamus, ChR2-eYFP labeled terminals in the amygdala were stimulated with 470-nm light pulses (1–3 ms) from a light-emitting diode (X-cite XLED, Lumen Dynamics) through the 20 × 1.0 NA objective of an upright microscope (Axio Examiner D1; Zeiss). In AMPA/NMDA experiments, pure glutamatergic components were isolated at the calculated chloride reversal potential of our internal solution (−70 mV), in presence of 20 μM Bicuculline-containing ACSF. NMDA currents were isolated in 20 μM Bicuculline and 20 μM NBQX. Miniature excitatory postsynaptic currents (mEPSCs) and miniature inhibitory postsynaptic currents were recorded in the same neuron by recording mEPSCs for 3 min at −70 mV (the chloride reversal) and then recording miniature inhibitory postsynaptic currents (mIPSCs) for 3 min at 0 mV (the cation – AMPA receptor mediated current reversal potential). In these experiments action potentials were blocked with TTX-citrate 2 μM and NMDA currents were blocked with 50 μM APV.

#### Long-Term Potentiation

Long-term potentiation was induced by stimulating the thalamic pathway to mpITCs with bipolar tungsten electrodes (World Precision Instruments, United States) using a protocol that has been validated at thalamic inputs onto LA principal cells (Humeau et al., 2007). The induction protocol consists of thalamic afferent (internal capsule) stimulation (six times 100 Hz; 1 s; 0.1 Hz) paired with postsynaptic depolarization to −20 mV. The experiment comprises a 5-minute baseline recording in current clamp with test pulses at 0.1 Hz, followed by 30 min post-induction recording under the same conditions. For statistical comparisons, LTP was quantified by normalizing the average EPSP amplitude during the last 3 min of post-induction sessions relative to the last 3 min of baseline.

Data were acquired with a Multiclamp 700B amplifier, Digidata1440, and Clampex software (Molecular Devices). Signals were filtered at 2 kHz, digitized at 5 kHz. Evoked synaptic responses were analyzed with NeuroMatic^1^ and custom-written macros in IgorPro (Wavemetrics). mEPSCs and mIPSCs were analyzed with Clampfit software (Molecular Devices) using the template search event detection algorithm with manual verification of each event.

All drugs were purchased from Tocris Bioscience (Bristol, United Kingdom).

### Confocal Imaging

Following electrophysiology experiments, slices containing injection sites and projections were fixed overnight in 4% paraformaldehyde. Slices were then washed three times (10 min each) in PBS, and mounted with Vectashield (Vector Laboratory Inc., Burlingame, CA, United States). Images were acquired using a laser-scanning microscope (LSM 780; Carl Zeiss Germany) with a 20 × 0.8 NA objective for injection sites.

### Statistics

All statistical analyses were performed using GraphPad Prism 7 (GraphPad Software, Inc, United States). Data are presented as mean ± SEM. ANOVA was performed to determine variance in experimental groups and Tukey’s *post hoc* correction was performed for multiple comparisons. For mEPSC and mIPSC cumulative frequency distribution analysis, the Kolmogorov–Smirnov test was used. Data were considered significant if *p* < 0.05. Significance levels are denoted as follows: ^∗^*p* < 0.05, ^∗∗^*p* < 0.01, ^∗∗∗^*p* < 0.001.

## Results

### ErbB4 Deletion From mpITCs Results in Enhanced mIPSC Amplitude

**FIGURE 1:**
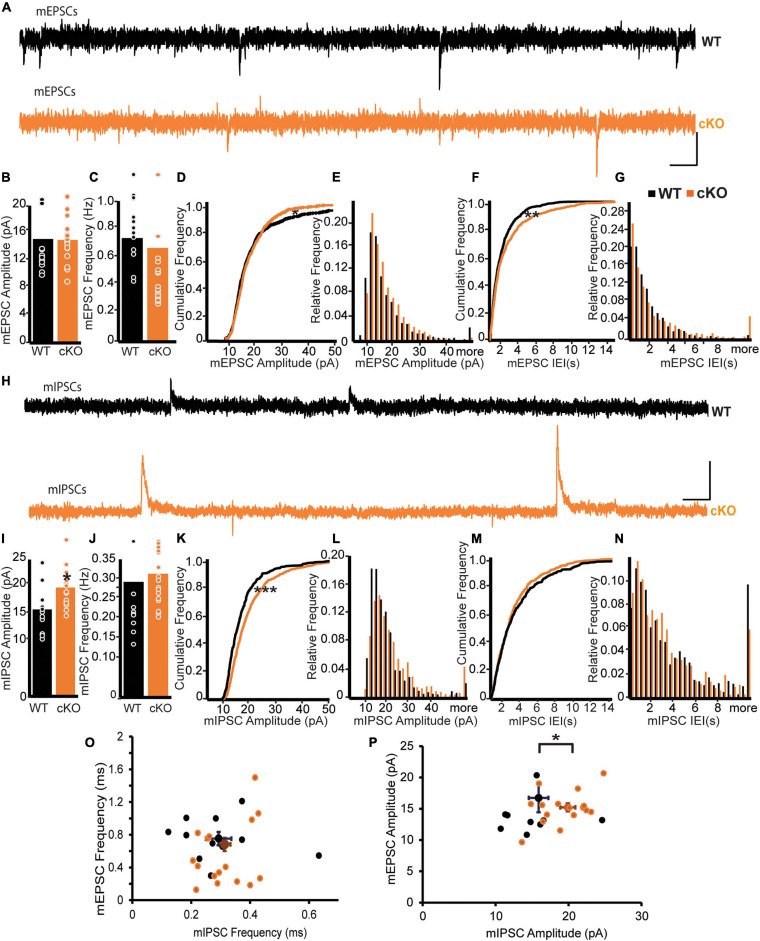
ErbB4 deletion from mpITCs results in enhanced mIPSC amplitude. **(A–G)** Properties of mEPSCs in WT (*n* = 13) and cKO mice (*n* = 18). **(A)** Sample traces of mEPSPs in WT (top, black) and cKO mice (bottom, orange). Scale bars: 10 pA, 0.2 s. **(B)** Bar graphs of mean mEPSC amplitudes and mEPSC frequencies. Panel **(C)** showing no difference between WT and cKO mice. *p* = 0.3088 and 0.7145 for amplitudes and frequencies, respectively. **(D)** Cumulative probability distributions of mEPSC amplitudes in WT and cKO. *p* = 0.0221. **(E)** Cumulative histogram summarizing the distribution of mEPSC amplitudes in WT and cKO. **(F)** Cumulative probability of mEPSC inter-event intervals (IEIs) in WT and cKO. *p* = 0.0052. **(G)** Cumulative histogram summarizing the distribution of mEPSC IEIs in WT and cKO. **(H–N)** Properties of mIPSCs in WT (*n* = 11) and cKO mice (*n* = 17). **(H)** Sample traces of IPSPs in WT (top, black) and cKO mice (bottom, brown). Scale bars: 10 pA, 0.1 s. **(I)** Enhanced mIPSC amplitudes in mpITCs recorded from cKO mice. *p* = 0.0200. **(J)** No difference in mean mIPSC frequencies between WT and cKO mice. *p* = 0.6202. **(K)** Cumulative probability of mIPSC amplitudes in WT and cKO. *p* < 0.0001. **(L)** Cumulative histogram summarizing the distribution of mIPSC amplitudes in WT and cKO. **(M)** Cumulative probability of mIPSC IEIs in WT and cKO. *p* = 0.2779. **(N)** Cumulative histogram summarizing the distribution of mIPSC IEIs in WT and cKO. **(O,P)** Analysis of mEPSCs and mIPSCs recorded at –70 mV (Cl^–^ reversal) and 0 mV, respectively, in the same cells. WT (*n* = 11), cKO mice (*n* = 17). mEPSC frequency/mIPSC frequency **(O)** were similar in WT and cKO mice. *p* = 0.2180; but mEPSC amplitude/mIPSC amplitude **(P)** were reduced in cKO mice. *p* = 0.011. Cumulative probability and mean data in WT and cKO mice were compared using KS-test and Student’s *t*-test, respectively. *p < 0.05; ***p < 0.001.

Functional connectivity of mpITCs is critical for the computational role of the amygdala in fear learning and extinction (Geracitano et al., 2012; Palomares-Castillo et al., 2012). To assess the effect of ErbB4 deletion on global synaptic drive to mpITCs, we recorded and analyzed miniature excitatory postsynaptic currents (mEPSCs) and mIPSCs in mpITCs in the presence TTX (Figures 1A–P). We found no difference in the mean mEPSC amplitude or frequency between WT and ErbB4 cKO mice (Figures 1B,C). However, analysis of the cumulative frequency of occurrence distributions for both the mEPSC amplitude and interevent event interval (IEI) revealed some differences in the distributions apparent in the cumulative plots and histograms (Figures 1D–G) (Kolgomov–Smirnov, mEPSC Amp *p* = 0.022, mEPSC IEI *p* = 0.0052). With respect to mEPSC amplitude the KO distribution had fewer small events and fewer large events. More smaller IEIs counter balanced by more large IEIs characterized the KO mEPSC IEI cumulative distribution. Although these changes in the distributions of mEPSCs were not apparent in the mean, they reflect the ensemble of AMPA currents from all excitatory inputs to mpITCs and it is possible that a subset of presynaptic inputs are more susceptible to ErbB4 loss than others. Although the mean frequency of mIPSCs was similar in WT and ErbB4 cKO mice, mIPSC amplitudes were significantly enhanced in ErbB4 cKO mice (Figures 1H–J). Cumulative frequency and histograms show the uniform shift in mIPSC amplitude to larger amplitudes (Figures 1K,L) and the lack of change in IEI (Figures 1M,N) (Kolgomov–Smirnov, mIPSC Amp *p* < 0.0001, mIPSC IEI *p* = 0.28). This indicates that aberrant inhibitory synaptic transmission is likely due to abnormal receptor composition or function at the postsynaptic sites of ErbB4 cKO mice. To evaluate the effect of ErbB4 deletion on the ratio of excitation to inhibition in individual mpITC neurons, we analyzed mEPSCs and mIPSCs recorded in the same neuron at −70 mV (chloride reversal potential) and 0 mV, respectively (Figures 1O,P). Indeed, there was a decreased mEPSC/mIPSC amplitude ratio due to specific increase in mIPSC amplitude in individual mpITC neurons in ErbB4 cKO mice (Figure 1P). This suggests that ErbB4 deletion reduces excitation-inhibition balance at the level of individual mpITCs.

### Thalamic Input-Driven Inhibition Onto mpITCs Is Enhanced in ErbB4 cKO Mice

**FIGURE 2:**
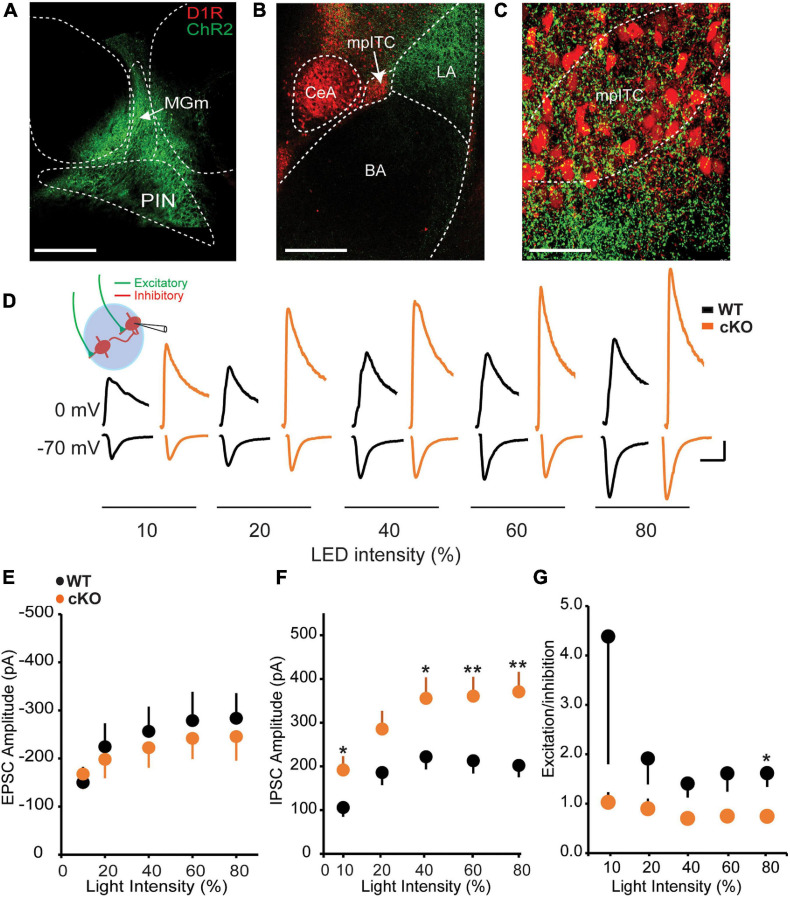
Thalamic input-driven inhibitory transmission onto mpITCs is enhanced in cKO mice. **(A)** ChR-eYFP injection site in PIN/MGm. **(B)** ChR [AAV-hsyn-hChR2(H134R)]-eYFP–labeled afferents in amygdala. **(C)** Labeled fibers in mpITC cluster. **(D)** Sample traces of EPSCs and IPSCs recorded at –70 and 0 mV, respectively, in mpITCs while stimulating thalamic fibers. Scale bar: 100 pA, 30 ms. No difference in mean EPSC amplitudes **(E)** [WT: *n* = 19, cKO mice *n* = 15; two-way ANOVA, *F*(1,158), *p* = 0.6522] but IPSC amplitudes **(F)** were significantly enhanced in cKO mice at various light intensities [WT: *n* = 19, cKO mice *n* = 15; two-way ANOVA, *F*(1,154), *p* < 0.0001]. **(G)** E/I ratio was significantly reduced in cKO mice. [WT: *n* = 19, cKO mice *n* = 15; two-way ANOVA, *F*(1,152), *p* = 0.0199.] *p < 0.05; **p < 0.01.

The inhibitory influence of mpITCs is driven in part by strong excitatory inputs from sensory thalamic areas such PIN and MGm, which convey auditory and somatosensory information to the amygdala during fear conditioning (Ehrlich et al., 2009; Asede et al., 2015). We therefore asked whether the observed enhancement of mIPSC amplitude is also reflected in evoked responses to PIN/MGm inputs onto mpITCs. To test this, ChR2-eYFP was transduced in PIN/MGm neurons resulting in visible ChR2-eYFP-labeled terminals in the LA and mpITC cluster (Figures 2A–C). Optogenetic activation of these terminals resulted in excitatory inputs and excitation-driven inhibition at −70 and 0 mV, respectively at various light intensities (Figure 2D). Although there was no difference in AMPA mediated EPSC amplitudes between WT and ErbB4 cKO mice, IPSC amplitudes were significantly enhanced in ErbB4 cKO mice (Figures 2E,F). This resulted in a corresponding decrease in excitation/inhibition (E/I) ratio in cKO mice (Figure 2G). Taken together, these results show that ErbB4 deletion alters spontaneous and sensory input-driven inhibitory transmission onto mpITCs.

### NMDA Receptor Hypofunction in ErbB4 cKO Mice

**FIGURE 3:**
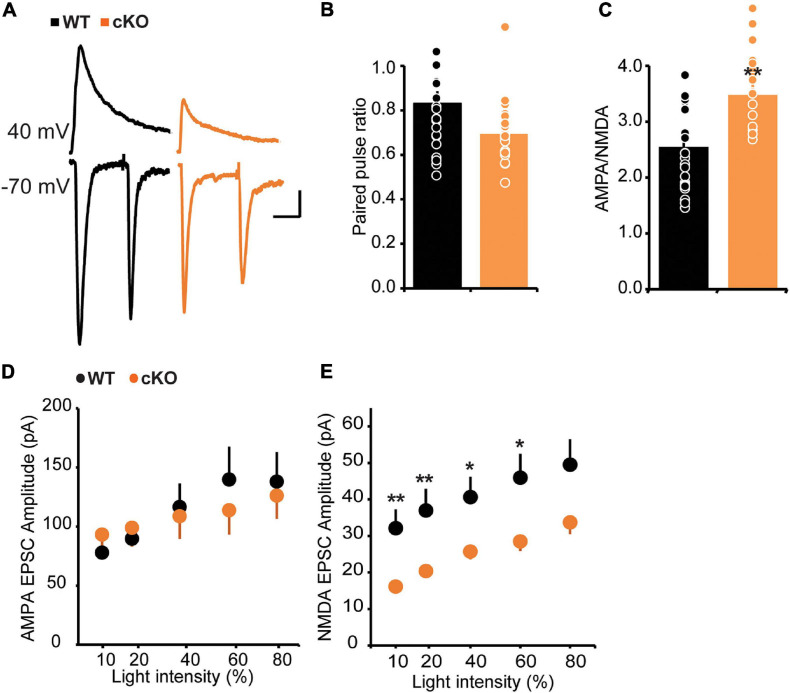
Increased AMPA/NMDA ratio and strength of NMDA receptor mediated synaptic transmission in cKO mice. **(A)** Sample traces of light evoked AMPA receptor EPSCs at –70 mV, and NMDA current recorded at +40 mV, 40 ms after stimulation onset. **(B)** No difference in PPR of AMPA receptor mediated EPSC between WT and cKO mice; *p* > 0.093 (*n* = 20 for both groups). **(C)** Increase in AMPA/NMDA ratio in cKO mice; *p < 0.05. **(D)** Input-output function of AMPA EPSCs is unaltered in cKO mice compared to WT [WT: *n* = 14, cKO *n* = 15; two-way ANOVA, *F*(1,130), *p* = 0.9315]. **(E)** Decreased NMDA EPSC amplitudes across stimulation intensities. [WT: *n* = 23, cKO *n* = 22; two-way ANOVA, *F*(1,210), *p* < 0.0001.] **p < 0.01.

In addition to the modulation of GABAergic function, ErbB4 also regulates glutamatergic transmission, especially that mediated by NMDA receptors (Siever and Davis, 2004; Fazzari et al., 2010; Del Pino et al., 2013). To test the effect of ErbB4 deletion on glutamatergic transmission onto mpITCs, we stimulated ChR2-labeled thalamic fibers with blue light in the presence of GABA_A_ receptor antagonist (20 μM Bicuculline, BIC) and analyzed paired pulse ratio (PPR) and AMPA/NMDA ratio as proxies for pre- and postsynaptic function, respectively (Figures 3A–C). We found no difference in the PPR between WT and ErbB4 cKO mice but the AMPA/NMDA ratio was significantly increased in ErbB4 cKO mice. This could be due to enhanced AMPA receptor or deficient NMDA receptor mediated synaptic transmission. To address this, we evaluated the input-output function of isolated AMPA or NMDA receptor mediated synaptic currents in separate experiments (Figures 3D,E). There was no difference in AMPA current amplitude between WT and ErbB4 cKO mice; however, the mean amplitude of NMDA receptor mediated currents was significantly reduced at various light stimulation intensities in ErbB4 cKO mice. This indicates that the increase in AMPA/NMDA ratio observed in ErbB4 cKO mice is due to reduced strength of NMDA receptor mediated synaptic transmission (Figures 3D,E).

### ErbB4 Deletion Impairs LTP of Thalamic Inputs Onto mpITCs

**FIGURE 4:**
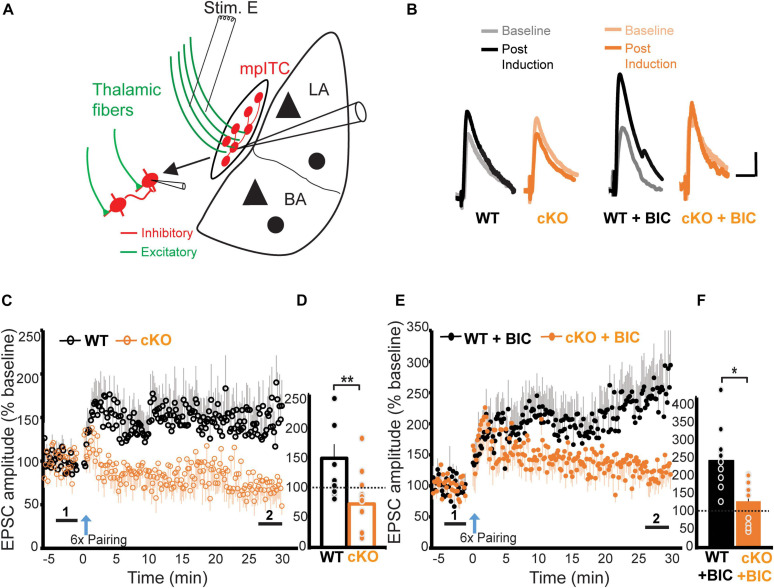
ErbB4 deletion impairs LTP of thalamic inputs to mpITCs. **(A)** A schematic diagram showing the placement of the stimulating electrode in the internal capsule to recruit thalamic fibers, while recording synaptic responses in mpITCs. **(B)** Sample traces of baseline (light) and post-induction (dark) EPSPs. Scale bars represent 3 mV, 30 ms. **(C)** Summary data showing that ErbB4 suppresses LTP of thalamic inputs to mpITCs in drug-free ACSF. **(D)** Quantification of **(C)**. Mean post-induction EPSP amplitude is decreased in cKO mice compared to WT mice in drug-free condition. WT vs. cKO, *p* = 0.008; cKO experienced long-term depression after induction (cKO vs. baseline, *p* = 0.032). Experimental groups: WT, *n* = 10; cKO; *n* = 10. WT 146.0 ± 23.0%, cKO 71.2 ± 13.0%; **(E)** Summary data showing ErbB4 suppression of LTP of thalamic inputs to mpITCs in the presence of inhibition blocker (bicuculline, BIC) in ACSF. **(F)** Quantification of **(E)**. Mean post-induction EPSP amplitude is decreased in cKO + BIC mice compared to WT + BIC mice in the presence of 20 μM BIC (WT + BIC vs. cKO + BIC, *p* = 0.010). Experimental groups: WT + BIC, *n* = 10; cKO + BIC; *n* = 11. WT + BIC 232.0 ± 37.4%, cKO + BIC 123.5 ± 17.7%. Mean data in **(D,F)** are averages of the last 3 min of post-induction recordings denoted by “2” in **(C,E)**. Dash line represents normalized baseline (1). *p < 0.05; **p < 0.01.

So far, our data shows that ErbB4 deletion from mpITCs results in enhanced inhibitory transmission and deficits in NMDA receptor mediated synaptic transmission. Because the ability to induce LTP is dependent on both inhibitory gating and NMDA receptor function, we hypothesized that ErbB4 might also be critical for LTP onto mpITCs. To test this, we examined LTP at thalamo-mpITC synapses by pairing presynaptic stimulation of thalamic afferents in the internal capsule with a pattern that reliably induces LTP at other synapses (1 s; 100 Hz; repeated six times at 0.1 Hz intervals) with postsynaptic depolarization to −20 mV (Humeau et al., 2007). With intact inhibition, this stimulation induced LTP at thalamo-mpITC synapse in WT mice (Figures 4A–F). Surprisingly, the same protocol induced long-term depression (LTD) in ErbB4 cKO mice, suggesting that the enhanced inhibition in combination with reduced NMDA receptor function in ErbB4 cKO mice reversed the direction of activity-dependent plasticity (WT 146.0 ± 23.0%, cKO 71.2 ± 13.0%, *p* = 0.008; Figures 4C,D). To isolate the potential effects of the observed NMDA receptor hypofunction on LTP, we compared LTP magnitude in WT and ErbB4 cKO mice in the presence of a GABA_A_ receptor antagonist, bicuculline (BIC). LTP of thalamo-mpITC synapses was significantly suppressed in ErbB4 cKO mice (WT + BIC 232.0 ± 37.4%, cKO + BIC 123.5 ± 17.7%, *p* = 0.010; Figures 4E,F). Taken together, ErbB4 regulates thalamic driven-inhibition and NMDA receptor mediated transmission, which are critical for LTP at thalamo-mpITC synapses.

## Discussion

This study provides novel evidence about the functional role of ErbB4 in mpITCs in amygdala circuits. ErbB4 deletion selectively enhanced spontaneous and thalamic-driven inhibition onto mpITCs resulting in an excitation-inhibition imbalance in ErbB4 cKO mice. Whereas AMPA receptor mediated synaptic transmission from thalamic afferents was unaltered by ErbB4 deletion, NMDA receptor mediated transmission was severely compromised, as evident in the increased AMPA/NMDA ratio and decreased amplitude of evoked NMDA current. These deficits in synaptic transmission were correlated with reduced LTP of thalamic inputs onto mpITCs in the absence of inhibition in ErbB4 cKO mice. Furthermore, stimuli that induced LTP in the presence of inhibition in WT caused LTD in ErbB4 cKO mice.

Previous work on the role of ErbB4 in regulating the function of BLA neurons reported that acute blockade of ErbB4 signaling with ErbB4 inhibitors (ecto-ErbB4 and AG1478) did not alter mIPSC amplitude onto BLA principal neurons but decreased the frequency significantly indicating that ErbB4 signaling plays a role in regulating GABA release (Lu et al., 2014; Bi et al., 2015). Similarly, analysis of mIPSCs onto hippocampal CA1 pyramidal neurons in mice with conditional knock out of ErbB4 in inhibitory neurons originating in the medial ganglionic eminence (Lhx6-Cre:ErbB4^FL^) showed a decrease in the frequency of mIPSCs in ErbB4 mutants compared to controls, with no change in the amplitude of mIPSCs (Del Pino et al., 2013). We found that ErbB4 deletion from mpITCs selectively enhanced the amplitude of mIPSCs, without affecting their frequency, which is in contrast to these studies, demonstrating cell type-specific regulation of inhibition by ErbB4.

Our data is in agreement with findings in MSNs of the nucleus accumbens core, in which, ErbB4 deletion selectively enhanced the amplitude of mIPSCs (Geng et al., 2017). This intriguing similarity between the effect of ErbB4 deletion in mpITCs and MSNs, and in contrast to that observed in BLA interneurons might be traceable to the shared developmental origin of ITCs and striatal MSNs (Kaoru et al., 2010). The authors further showed that the enhancement in inhibitory transmission in MSNs following ErbB4 deletion was due to elevated GABA_A_ receptor α1 subunit expression. ErbB4 has been shown to associate with GABA_A_ receptor α1 but not α2 in a NRG2 dependent manner decreasing synaptic clustering of the receptor (Mitchell et al., 2013). Compelling evidence from electrophysiological and immunocytochemical experiments have showed that the α2 and α3, but not the α1 subunits of the GABA_A_ receptor were present at Imp cell (mpITC) synapses of the mouse amygdala (Geracitano et al., 2012). This raises the question of which GABA_A_ receptor subunit mediates the enhancement in inhibitory transmission to mpITCs found in ErbB4 cKO mice. Ultrastructural and functional analysis of GABA_A_ receptor subunit composition in mpITCs of ErbB4^F/F^:Drd1a^Cre(EY266)^ mice should ultimately resolve this issue.

The aberrant inhibitory transmission in mpITCs was further supported by an even more pronounced enhancement of thalamic-driven feedforward inhibition onto mpITCs of ErbB4 cKO. The fact that these evoked disynaptic inhibitory currents were enhanced considerably more than the mIPSCs may be the result of increased action potential mediated GABA release from the presynaptic terminal in the cKO. However, the lack of change in mIPSC frequency constrains potential mechanisms to processes that require depolarization of the terminal. Functional interconnectivity of mpITCs, is required to maintain the stability of firing patterns which is critical for their computational role in the amygdala (Geracitano et al., 2007). Therefore, polysynaptic disinhibitory microcircuits among mpITCs could further compound the enhanced thalamic-driven feedforward inhibition. It is unlikely that enhanced excitatory drive from thalamic inputs contributes to the enhancement given there was no change in AMPA current and reduced NMDA current amplitude.

Hypofunction of the NMDA receptor is hypothesized to be a mechanism underlying cognitive dysfunction in individuals with schizophrenia (Pitcher et al., 2011) and altered NRG1-ErbB4 signaling may contribute to NMDA receptor hypofunction seen in these patients (Hahn et al., 2006). We found that ErbB4 deletion from mpITCs significantly increased AMPA/NMDA ratio due to a reduction in the amplitude of NMDA receptor-mediated currents at thalamo–mpITC synapses. In agreement with our results, in paired recordings from layer V/VI pyramidal neurons onto fast spiking basket cells, the AMPA/NMDA ratio was increased due to a reduction in NMDA currents (Yang et al., 2013). Given that ErbB4 interacts with NMDA receptors through association with the postsynaptic scaffolding protein, PSD-95 (Garcia et al., 2000; Huang et al., 2000), which is important for stabilizing the NMDA receptor at the synapse, the reduction in NMDA current we observe may be related to the absence of this association in the ErbB4 cKOs. In the hippocampus and prefrontal cortex, NRG1 signaling through its receptor ErbB4 suppresses NMDAR function by preventing Src-mediated enhancement of NMDAR responses further supporting the role of ErbB4 in regulating NMDA receptors (Pitcher et al., 2011).

There was no difference in the strength of thalamic evoked AMPA currents onto mpITCs and no change in AMPA mEPSC frequency or amplitude. Our lack of regulation of AMPA currents by ErbB4 deletion is in contrast to the reduction in AMPA current in studies where ErbB4 is deleted in parvalbumin expressing interneurons in cortical structures such as hippocampus (Fazzari et al., 2010; Del Pino et al., 2013) and prefrontal cortex (Yang et al., 2013). Our results also differ from the increase in AMPA currents with ErbB4 deletion in other inhibitory nuclei studied such as the somatostatin expressing neurons of the thalamic reticular nucleus (Ahrens et al., 2015) and central lateral nucleus of the amygdala (Ahrens et al., 2018). Our observed selective reduction in NMDA current with no change in AMPA current has been previously observed when other key postsynaptic density proteins such as the NLGNs are deleted (Chen L. Y. et al., 2017; Wu et al., 2019). Together, the evidence highlights regional and cell-type specific roles of ErbB4-NRG signaling in the CNS.

Previous studies have demonstrated LTP of BLA inputs to mpITCs (Huang et al., 2014; Strobel et al., 2015). To the best our knowledge, our study is the first to demonstrate LTP at thalamo–mpITC synapses. We further showed that ErbB4 deletion suppresses this LTP likely via exaggerated inhibition and dampening of NMDA receptor function. Indeed, LTP at thalamic-amygdala synapses is suppressed and difficult to induce in naïve slices with intact inhibition (Bissiere et al., 2003; Shaban et al., 2006). In the BLA, NRG1-ErbB4 signaling is at saturated levels, which maintains high GABAergic activity. Therefore, neutralizing endogenous NRG1 or pharmacological or genetic ablation of ErbB4 in PV neurons reduces GABAergic transmission and enables LTP at thalamo-BLA principal cell synapses (Lu et al., 2014). mpITCs are interconnected and reciprocally connected to BLA principal cells (Geracitano et al., 2007; Asede et al., 2015). Therefore, the enhanced inhibition in mpITCs following ErbB4 deletion could dampen their output to BLA principal cells thereby facilitating LTP at thalamo-BLA principal cell synapses, similar to the effect observed in PV-ErbB4−/− mice.

Our data provide novel evidence that ErbB4 in mpITCs is critical for excitation-inhibition balance, NMDA receptor-mediated transmission, LTP at sensory thalamo-mpITC synapses.

## Data Availability Statement

The raw data supporting the conclusions of this article will be made available by the authors, without undue reservation.

## Ethics Statement

The animal study was reviewed and approved by the National Institute of Health under the approval of the Institutional Care and Use Committee of Max Planck Florida Institute for Neuroscience.

## Author Contributions

DA and MB conceived the project and wrote the manuscript. DA, JO, SA, and DD performed the experiments and analyzed the data. All authors contributed to the article and approved the submitted version.

## Conflict of Interest

The authors declare that the research was conducted in the absence of any commercial or financial relationships that could be construed as a potential conflict of interest.

## Publisher’s Note

All claims expressed in this article are solely those of the authors and do not necessarily represent those of their affiliated organizations, or those of the publisher, the editors and the reviewers. Any product that may be evaluated in this article, or claim that may be made by its manufacturer, is not guaranteed or endorsed by the publisher.

## Footnotes

1
http://www.neuromatic.thinkrandom.com
